# Curcumin Mitigates Muscle Atrophy Potentially by Attenuating Calcium Signaling and Inflammation in a Spinal Nerve Ligation Model

**DOI:** 10.3390/cimb46110742

**Published:** 2024-11-05

**Authors:** Casey Appell, Nigel C. Jiwan, Chwan-Li Shen, Hui-Ying Luk

**Affiliations:** 1 Department of Kinesiology and Sport Management, Texas Tech University, Lubbock, TX 79406, USA; casey.appell@ttu.edu (C.A.); jiwan@hope.edu (N.C.J.); 2 Department of Kinesiology, Hope College, Holland, MI 49423, USA; 3 Department of Pathology, Texas Tech University Health Sciences Center, Lubbock, TX 79430, USA; leslie.shen@ttuhsc.edu

**Keywords:** muscle mass, denervation, calcium signaling, inflammation, spinal nerve injury

## Abstract

Denervation-induced calcium/calmodulin-dependent protein kinase II (CaMKII) activation and inflammation can result in muscle atrophy. Curcumin and bisdemethoxycurcumin are well known to exhibit an anti-inflammatory effect. In addition, curcumin has been shown to attenuate CaMKII activation in neuronal cells. This study aimed to examine the effect of curcumin or bisdemethoxycurcumin on CaMKII activation, inflammation, and muscle cross-sectional area (CSA) in spinal nerve ligated rats. Sixteen female rats were assigned to sham (CON), spinal nerve ligation (SNL), SNL+ curcumin 100 mg/kg BW (100CUR), and SNL+ bisdemethoxycurcumin 50 mg/kg BW (50CMO) for 4 weeks. Ipsilateral (surgical) soleus and tibialis anterior (TA) muscles was stained for dystrophin to measure CSA. Ipsilateral and contralateral (non-surgical) plantaris muscles were analyzed for protein content for acetylcholine receptor (AChR), CaMKII, CaMKII^Thr286^, nuclear factor-κB (NF-κB), NF-κB^Ser536^, and interleukin-1β (IL-1β) and normalized to α-tubulin and then CON. A significant (*p* < 0.050) group effect was observed for TA CSA where CON (11,082.25 ± 1617.68 μm^2^; *p* < 0.001) and 100CUR (9931.04 ± 2060.87 μm^2^; *p* = 0.018) were larger than SNL (4062.25 ± 151.86 μm^2^). In the ipsilateral plantaris, the SNL (4.49 ± 0.69) group had greater CaMKII activation compared to CON (1.00 ± 0.25; *p* = 0.010), 100CUR (1.12 ± 0.45; *p* = 0.017), and 50CMO (0.78 ± 0.19; *p* = 0.009). The ipsilateral plantaris (2.11 ± 0.66) had greater IL-1β protein content than the contralateral leg (0.65 ± 0.14; *p* = 0.041) in the SNL group. In plantaris, the SNL (1.65 ± 0.51) group had greater NF-κB activation compared to CON (1.00 ± 0.29; *p* = 0.021), 100CUR (0.61 ± 0.10; *p* = 0.003), 50CMO (0.77 ± 0.25; *p* = 0.009) groups. The observed reduction in Ca^2+^ signaling and inflammation in type II plantaris muscle fibers might reflect the changes within the type II TA muscle fibers which may contribute to the mitigation of TA mass loss with curcumin supplementation.

## 1. Introduction

Denervation resulting in muscle atrophy [1,2,3] affects approximately 76 million individuals over the age of 40 (~14%) in the United States [4]. Lumbar region ligation (spinal nerve ligation {SNL}) has been used as a model of denervation [5,6,7,8,9]. Spinal nerve ligation (SNL)-induced denervation leads to calcium/calmodulin-dependent protein kinase II (CaMKII) activation [10] and a pro-inflammatory state [11,12,13]. Both CaMKII activation and a pro-inflammatory state can lead to muscle atrophy in denervated muscle [14,15,16,17]. Studies have shown that inhibiting CaMKII activation or inflammatory signaling prevents denervation-induced muscle atrophy [18,19]. Turmeric bioactive compounds, particularly curcumin and bisdemethoxycurcumin, are known for their anti-inflammatory properties [20,21,22,23] and have shown potential in reducing muscle atrophy in nerve damage models [24,25]. Growing evidence has shown that the polyphenol constituent of turmeric (i.e., curcumin) inhibits CaMKII activation in neuronal cells [26]. Given the importance of CaMKII activation and inflammatory signaling in SNL-induced atrophy [18,19], a gap in knowledge remains on the effect of curcumin on CaMKII and NF-κB signaling in denervated muscles.

SNL-induced denervation increases sarcoplasmic Ca^2+^ [17]. The increased sarcoplasmic Ca^2+^ has been attributed to the integration of highly Ca^2+^ permeable acetylcholine receptors (AChR) [27,28,29,30] into the neuromuscular junction region of the sarcolemma [31]. The increased sarcoplasmic Ca^2+^ rapidly activates CaMKII (CaMKII^Thr286^) [10,18], which can have various effects including contractile activity-dependent, fiber type-specific gene expression, and oxidative enzyme expression regulation [32]. In support of this mechanism, Castets et al. reported that 1 week following denervation, CaMKII activation was greater in the ipsilateral (i.e., surgical) tibialis anterior (TA) muscle compared to the contralateral (i.e., non-surgical) leg [10]. CaMKII activation has been shown to activate nuclear factor-kappa B (NF-κB) by up-regulating calcineurin (a downstream target of CaMKII) leading to up-regulation of the p65 subunit of NF-κB in neuronal cells [33]. However, up-regulation of NF-κB by CaMKII activation, to our knowledge, has not been investigated in muscle cells. Denervation has been shown to increase NF-κB activation [11,12,13] and has been shown to modulate denervation-induced muscle atrophy associated with increased reactive oxygen species (ROS) production [19]. ROS are highly reactive molecules containing oxygen (e.g., superoxide anion, hydrogen peroxide, hydroxyl radical), which in excess can damage cellular components and lead to muscle atrophy [34]. Considering that inhibition of either CaMKII and NF-κB activation attenuates denervation-induced muscle atrophy [18,19], it is important to investigate CaMKII or anti-inflammatory targeted interventions to attenuate muscle atrophy in nerve damage models.

The rhizomes of turmeric (*Curcuma longa* L.), a plant of the *Zingiberaceae* family, contain three different curcuminoids which have been attributed to turmeric’s pharmacological activity. These curcuminoids include curcumin [1,7-bis(4-hydroxy-3-methoxyphenyl)-1,6-heptadiene-3,5-dione], demethoxycurcumin [1-(4-hydroxyphenyl)-7-(4-hydroxy-3-methoxyphenyl)-1,6-heptadiene-3,5-dione], and bisdemethoxycurcumin [1,7-bis(4-hydroxyphenyl)-1,6-heptadiene-3,5-dione] [35,36]. Curcumin has two symmetric *o*-methoxy phenols linked through the α,β-unsaturated β-diketone moiety. In contrast to curcumin, bisdemethoxycurcumin is deficient in two *o*-methoxy substitutions, and demethoxycurcumin has one *o*-methoxy substitution [37]. The difference in chemical structure among these curcuminoids gives rise to diverse biological activities, altering ROS scavenging capacity and interactions with various molecular targets including proteins, DNA, lipids, metals, and metalloproteins [38].

Curcumin has been used to alleviate symptoms of denervation (e.g., peripheral neuropathic pain) [24,39,40] and attenuate TA muscle mass loss in nerve damage-induced denervation models [24,25]. While the mechanism of curcumin on muscle mass loss has not been explored, curcumin dose-dependently attenuated CaMKII activation in neuronal cells [26,41,42,43]. In addition, curcumin attenuated NF-κB activation concomitant with a lower IL-1β protein content in asthma and allergen-induced pro-inflammatory lung tissue [44,45] and lipopolysaccharide-induced pro-inflammatory mastitis [46]. Similar to curcumin, bisdemethoxycurcumin has been shown to reduce pro-inflammatory signaling (i.e., reduce NF-κB expression) in renal and immune cells [47,48]. However, in comparison to curcumin, bisdemethoxycurcumin suppressed NF-κB activity to a lesser extent [49]. Santosh et al. speculated that the phenyl methoxy groups in curcumin play a critical role in suppressing NF-κB activation which are absent in bisdemethoxycurcumin [49]. While bisdemethoxycurcumin may be less active for certain biomolecular targets, it is more active for others [37]. For example, in comparison to curcumin, bisdemethoxycurcumin is a more active antioxidant (i.e., attenuating superoxide production) [50] as well as having greater chemical stability [51] and bioavailability [52].

Acknowledging the role of CaMKII and NF-κB activation in SNL-induced muscle mass loss [14,15,16,17], curcumin’s and bisdemethoxycurcumin’s capacity to attenuate CaMKII (curcumin only) and NF-κB activation could mitigate muscle mass loss in SNL models. However, to our knowledge, no study has investigated the effect of curcumin or bisdemethoxycurcumin on CaMKII activation, inflammatory signaling, and muscle mass loss following denervation. To address this gap, the main objective of the current study is to investigate the effect of curcumin or bisdemethoxycurcumin on CaMKII and NF-κB activation, IL-1β, and muscle cross-sectional area (CSA) in SNL rats.

## 2. Materials and Methods

### 2.1. Animals

A total of 16 female Sprague-Dawley rats (150–180 g body weight, Envigo; Cumberland, VA, USA) and housed individually under a 12 h light–dark cycle with food and water ad libitum. Body weights, food intake, and water consumption were recorded weekly. All conditions and handling of the animals were approved by the Texas Tech University Health Sciences Center Institutional Animal Care and Use Committee (IACUC # 21007). All experiments were performed according to the relevant guidelines and regulations.

### 2.2. Spinal Nerve Ligation Procedure

After 5 days of acclimatization, 12 animals were randomly assigned to undergo spinal nerve ligation procedures, while the remaining 4 animals underwent a sham surgery procedure (CON). The spinal nerve ligation procedure was used to induce denervation in the ipsilateral hind paw, as described previously [53,54]. Isoflurane was used for induction (3%) and maintenance (2%) of anesthesia throughout the procedure. After removing the L5/L6 level paraspinal muscles and underlying L6 transverse process, the L5 spinal nerve was removed from adjacent structures and securely ligated with 6–0 silk thread. The paraspinal muscles were closed with sutures, and the skin was clipped together. Sham-operated animals served as controls, receiving the same surgical steps of spinal nerve ligation procedure without ligation of the L5 spinal nerve. After surgery, all animals were given antibiotic treatment (1 dose of gentamycin, 8 mg/kg, subcutaneously, s.c.; VetOne, Boise, ID, USA) and were monitored for any signs of infection or distress. Throughout the study period, the animals were monitored to reduce unnecessary stress or pain following ethical guidelines of the International Association for the Study of Pain [55].

### 2.3. Dietary Treatments

The 16 female Sprague-Dawley rats were randomly assigned to four groups: sham (CON), spinal nerve ligation (SNL), SNL + 100 mg curcumin C3 complex^®^/kg BW (100CUR), and SNL + 50 mg bisdemethoxycurcumin/kg BW (50CMO). Curcumin C3 complex^®^ (CUR) is a standardized extract containing a ratio-defined mixture of three curcuminoids (77.3% curcumin, 19.0% demethoxycurcumin, and 3.7% bisdemethoxycurcumin) that achieved the GRAS (Generally Recognized as Safe) status [56]. CMO was a >99% purity product from turmeric extract. Both CUR and CMO were a gift from Sabinsa Corporation (East Windsor, NJ, USA). All animals were given AIN-93G diet (Research Diet, Inc., New Brunswick, NJ, USA; D10012G). For the 100CUR and 50CMO groups, after the SNL procedure, CUR and CMO supplements were given in the AIN-93G diet for 4 weeks, respectively. Body weight, food intake, and water consumption were recorded weekly.

In addition to CUR, we selected to test purified bisdemethoxycurcumin (CMO) to compare it with CUR as CMO has been shown to be more chemically stable [51] and bioavailable [52] than curcumin. In addition, bisdemethoxycurcumin has been shown to reduce pro-inflammatory signaling (i.e., reduce NF-κB expression) in renal and immune cells [47,48]. Bisdemethoxycurcumin’s multi-mechanistic mode of action would enable its potential efficacy in treating spinal nerve ligation-induced muscle atrophy. Previously published data from the same study assessing pain using 3 different doses of CUR (25, 50, and 100 mg/kg BW) and CMO (50 mg/kg BW) were used to select the groups to evaluate their effects on CaMKII activation, inflammation, and muscle cross-sectional area (CSA) [57]. Briefly, Electronic von Frey Aesthesiometer (IITC Life Science, Woodland Hills, CA, USA) was used to evaluate the left hind paw mechanical withdrawal thresholds of spinal nocifensive reflexes. Testing was conducted in a dedicated testing area at baseline (week 0) and weekly for four weeks. Among all groups, only CUR at 100 mg/kg BW and CMO at 50 mg/kg BW had significantly decreased hypersensitivity relative to the untreated SNL group. The effect was observed as early as 1 week after starting CUR or CMO [57]. Therefore, we selected CUR at 100 mg/kg BW and CMO at 50 mg/kg BW for the current study.

### 2.4. Sample Collection

After 4 weeks, the animals were anesthetized with isoflurane gas before being euthanized by CO_2_ gas inhalation. Ipsilateral (surgical, right) soleus, ipsilateral TA, ipsilateral plantaris, and contralateral (non-surgical, left) plantaris muscles were collected from the rats after being fasted for 4 h and were stored at −80 °C for further analyses. Soleus and TA muscles were cleaned of adhering connective tissues, and frozen in the Tissue-Tek optimal cutting temperature (O.C.T.) compound for further cross-sectional area analysis. Due to limited TA muscle samples, plantaris muscle samples were collected as a replacement as both muscles are composed primarily of type II muscle fibers [58]. Ipsilateral and contralateral plantaris muscles were cleaned of adhering connective tissues and were flash frozen in liquid nitrogen for further protein analysis. The contralateral plantaris was compared to the ipsilateral plantaris as the spinal nerves that innervate the contralateral leg are anatomically distinct from the ipsilateral and are not directly affected by the ligation of the L5 nerve on the ipsilateral side [59].

### 2.5. Cross-Sectional Area (CSA) Analysis

Previously frozen whole soleus and TA muscle samples in O.C.T. compound were sectioned (10 μm thick) at −25 °C in a cryostat and placed on positively charged microscope slides (Fisher Scientific, Waltham, MA, USA; 22-037-246). CSA was visualized using dystrophin immunohistochemistry staining as described previously [60]. Muscle cross-sections were rehydrated in PBS for 10 min at room temperature. Next, the slides were washed to remove excess O.C.T. and incubated at room temperature with H_2_O_2_ for 10 min. Next, the slides were washed and incubated with blocking solution (1% bovine serum albumin in PBS) for 20 min. Following this, the slides were washed and incubated with primary antibodies directed against dystrophin (1:1000; Thermofisher, Waltham, MA, USA; Pa5-32388). Thereafter, the slides were incubated in biotinylated horse anti-mouse IgG (1:500; Thermofisher, Waltham, MA, USA; 31806) diluted in PBS for 2 h. After a final wash, the slides were mounted using DAPI mounting media with cover glasses and let incubate for 20 min before imaging. The slides were then visualized using a Zeiss Axiovert 200 m Inverted Fluorescent Motorized Microscope (Ziess, Oberkoche, Germany). Images were captured and muscle CSA was analyzed using Image J (Version 1.54g, National Institute for Health, Bethesda, MD, USA) for all samples. One hundred muscle fiber areas were measured for each sample.

### 2.6. Western Blot Analysis

As whole TA muscles were stored in O.C.T compound and utilized for CSA analysis, plantaris muscles were chosen as a substitute being that both muscle groups are predominantly composed of type II muscle fibers [58]. A detailed description of muscle homogenization and Western blot analyses have been published previously [60]. Frozen plantaris muscle samples (~30 mg) were combined with 200 μL/mg muscle of ice-cold tissue extraction reagent I (Thermofisher, Waltham, MA, USA; FNN0071) containing 1× protease and phosphatase inhibitor (1:100; Sigma Aldrich, St. Louis, MO, USA; 78442). Next, 3 glass beads (1 mm) were added to each sample and homogenized using a FastPrep Tissue Homogenizer (MP Biomedicals, Santa Ana, CA, USA) set to 6.5 m/s for 120 s on the QuickPrep adaptor setting. Homogenized muscle samples were agitated at 4 °C, then centrifuged at 15,000× *g* at 4 °C for 20 min. The supernatant was collected and analyzed for total protein concentration by BCA protein assay reagent (Pierce, Rockford, IL, USA; A55865) according to manufacturer instructions. Absorbance was measured at 562 nm using microplate spectrophotometry (Bio-Tek, Winooski, VT, USA). Samples were stored at −80 °C until protein analysis.

Aliquots of total protein from each sample were diluted in 2 Laemmli buffer (Bio-Rad, Hercules, CA, USA; 1610737) with 1 M dithiothreitol (Bio-Rad, Hercules, CA, USA; 1610610). SDS-PAGE was used to separate total protein according to molecular weight (120 V; 4–20% Mini-PROTEAN TGX gel, Bio-Rad, Hercules, CA, USA; 4561094). The ipsilateral and contralateral plantaris muscle from 1 animal per group (a total of 4 animals) were loaded on the same gel to account for variability among gels. Protein was then transferred (70 V at 4 °C) to PVDF (Bio-Rad, Hercules, CA, USA; 1620184) membranes for immunoblotting. To control for inter-membrane variability, duplicate horizontal sections were transferred onto the same PVDF membrane. PVDF membranes were blocked in 5% *w*/*v* nonfat dry milk in TBS containing 0.1% Tween-20 (TBST) and room temperature for 1 h. Next, PVDF membranes were incubated overnight at 4 °C with primary antibodies against AChR subunit CHRNA1 (1:1000; Thermofisher, Waltham, MA, USA; MA3-043), CaMKII (1:1000; Cell Signaling, Danvers, MA, USA; 4436S), CaMKII^Thr286^ (1:1000; Cell Signaling, Danvers, MA, USA; 12716S), NF-κB (1:1000; Cell Signaling, Danvers, MA, USA; 8242S), NF-κB^Ser536^ (1:1000; Cell Signaling, Danvers, MA, USA; 3033), IL-1β (1:1000; RandDSystems, Minneapolis, MN, USA; MAB5011), and α-tubulin (1:4000; Cell Signaling, Danvers, MA, USA; 3873S). Primary antibodies were diluted in TBST with 3% nonfat dry milk. Subsequently, PVDF membranes were incubated for 1 h at room temperature with either secondary anti-mouse IgG (1:5000; Cell Signaling, Danvers, MA, USA; 7076S) or anti-rabbit IgG (1:5000; Cell Signaling, Danvers, MA, USA; 7074S), respectively. Stained protein bands were visualized using a chemiluminescent substrate (Advanta Inc., San Jose, CA, USA; K-12043-D10), and the ChemiDoc MP Imaging System (BioRad Laboratories, Hercules, CA, USA) was used to picture the stained protein bands. Membranes were stripped using 5× Western Reprobe (G-Biosciences, St. Louis, MO, USA; 786-306) for 90 min and reblotted for antibodies. Image Lab Software 6.1 (BioRad Laboratories, Hercules, CA, USA; 12003154) was used to analyze band density. Total protein concentrations were normalized to a housekeeping protein α-tubulin then to the CON group and expressed as arbitrary units. To analyze the activation state of CaMKII and NF-κB, the phosphorylated to total protein ratio, normalized to α-tubulin (i.e., CaMKII ^Thr286^/CaMKII/α-tubulin, NF-κB^Ser536^/NF-κB/α-tubulin) was calculated. See Supplementary Materials (Figure S1) for full Western Blot images.

### 2.7. Statistical Analysis

SPSS (IBM version 29; Armonk, NY, USA: IBM Corp.) was used for all statistical analyses. Data for each variable was evaluated to determine if the assumptions (e.g., normality, sphericity) for parametric statistics were met. Log10 transformation was used if the assumption of parametric statistics (e.g., normality, sphericity) were violated. The cross-sectional area was analyzed using a one-way ANOVA (group) while protein content was analyzed using a two-way ANOVA (group × leg), and Fisher’s least significant difference post hoc test was used for pairwise comparisons. The statistical significance was set at *p* < 0.050. Data are reported as mean ± SEM.

## 3. Results

### 3.1. Muscle Fiber Cross-Sectional Area (CSA)

**Figure 1:**
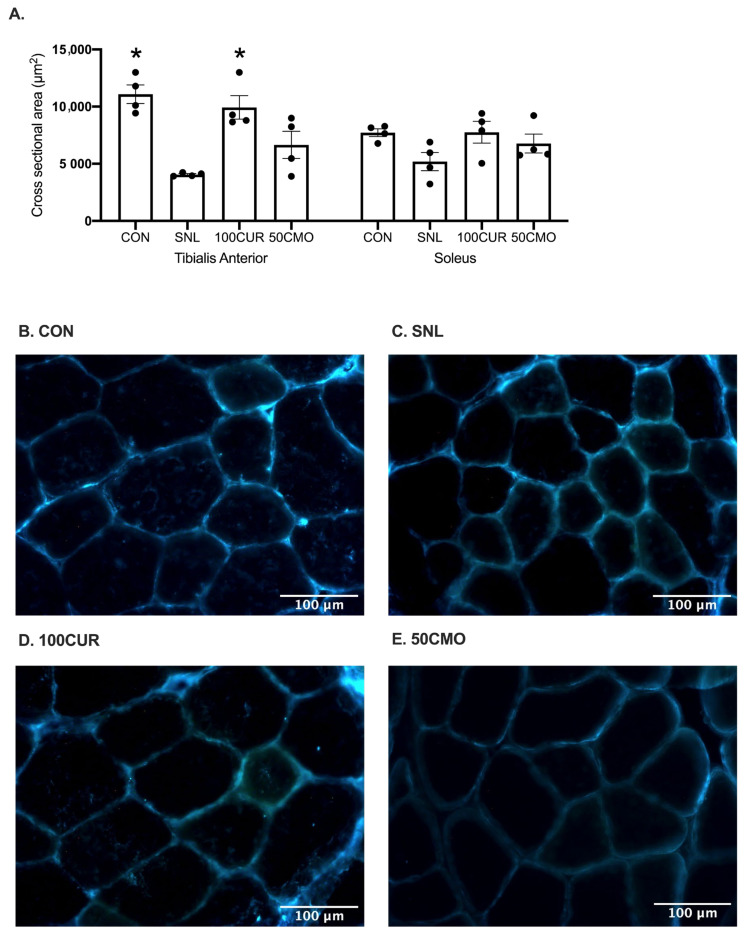
TA and soleus muscle fiber cross-sectional area (μm^2^) (**A**). Average number of muscle fibers (*n* = 100) counted per rat. Values are mean ± SEM (CON: *n* = 4; SNL: *n* = 4; 100CUR: *n* = 4; 50CMO: *n* = 4). * *p* < 0.050 vs. SNL. Representative microscopy images of the tibialis anterior muscle stained with dystrophin from CON (**B**), SNL (**C**), 100CUR (**D**), and 50CMO (**E**) groups.

For tibialis anterior (TA) muscle fiber CSA, a significant (*p* < 0.050) group effect was observed in the ipsilateral leg. Muscle fiber CSA in the CON (11,082.25 ± 1617.68 μm^2^; *p* < 0.001) and the 100CUR (9931.04 ± 2060.87 μm^2^; *p* = 0.018) groups were larger than the SNL (4062.25 ± 151.86 μm^2^) group (Figure 1). Additionally, no significant (*p* ≥ 0.050) differences were observed among CON, 100CUR, and 50CMO groups in the TA muscle. For soleus muscle, no significant differences in muscle fiber CSA were observed among groups.

### 3.2. Protein Analysis

#### 3.2.1. Acetylcholine Receptor (AChR)

No significant differences were observed for AChR protein content between the ipsilateral leg and the contralateral leg in all groups in the plantaris muscle.

#### 3.2.2. Calcium/Calmodulin-Dependent Protein Kinase II (CaMKII)

**Figure 2:**
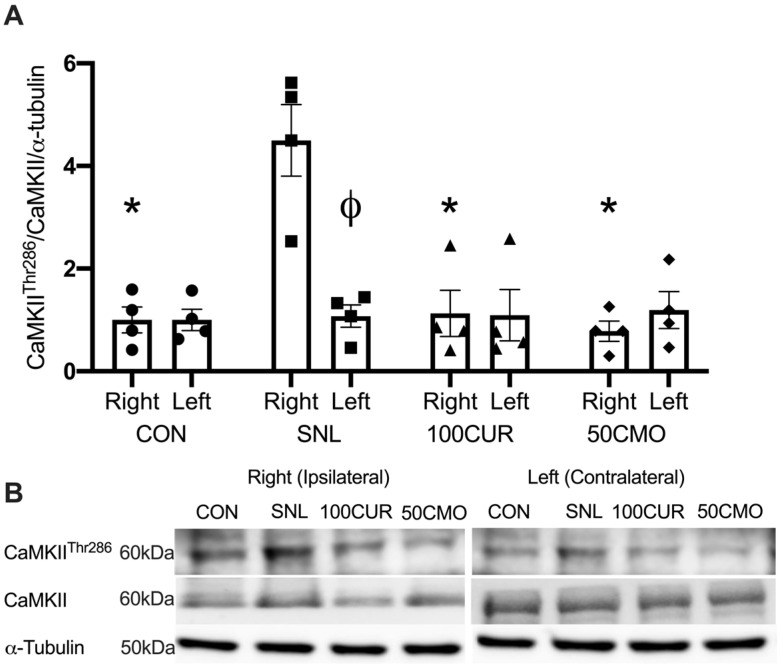
Plantaris protein content calculated results for calcium/calmodulin-dependent protein kinase II (CaMKII)^Thr286^/CaMKII/α-tubulin ratio (**A**). Data were normalized to CON. Values are mean ± SEM (CON: *n* = 4; SNL: *n* = 4; 100CUR: *n* = 4; 50CMO: *n* = 4). * *p* < 0.050 vs. SNL ipsilateral leg. Φ *p* < 0.050 vs. ipsilateral leg. The representative blot images display CaMKII^Thr286^, CaMKII and, α-tubulin for CON, SNL, 100CUR, and 50CMO groups (**B**). MW, molecular weight (kDa).

A significant group × leg interaction effect was observed for CaMKII^Thr286^/CaMKII/α-tubulin (i.e., CaMKII activation) in the plantaris. In the ipsilateral plantaris, the SNL (4.49 ± 0.69) had greater CaMKII activation compared to the CON (1.00 ± 0.25; *p* = 0.010), the 100CUR (1.12 ± 0.45; *p* = 0.017), and the 50CMO (0.78 ± 0.19; *p* = 0.009) groups (Figure 2). In addition, the ipsilateral leg (4.49 ± 0.69) had greater CaMKII activation than the contralateral leg (1.07 ± 0.21; *p* = 0.022) in the SNL group, whereas no significant differences were observed between ipsilateral and contralateral leg in the CON, the 100CUR, and the 50CMO group. No significant differences were observed for CaMKII protein content between the ipsilateral leg and the contralateral leg in all groups in the plantaris muscle.

#### 3.2.3. Nuclear Factor-κB (NF-κB)

**Figure 3:**
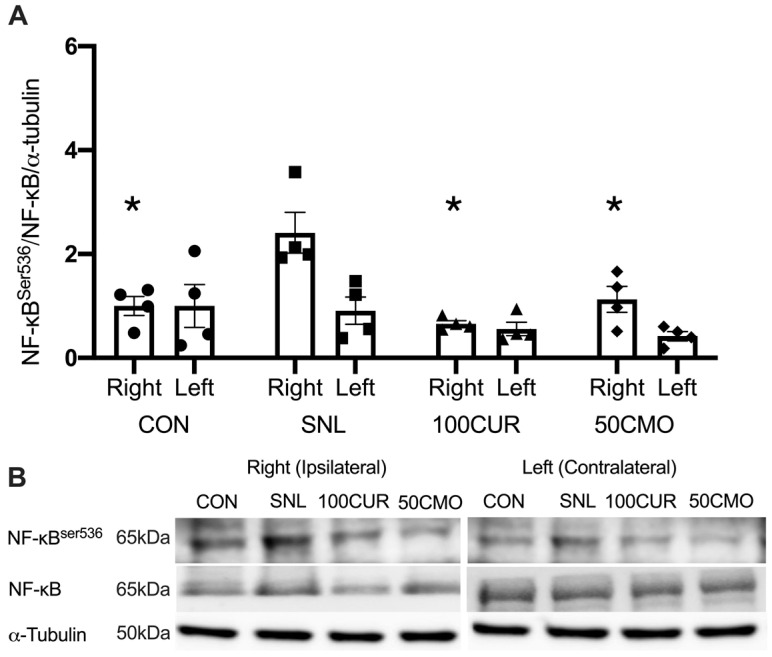
Plantaris protein content calculated results for nuclear factor-κB (NF-κB)^Ser536^/NF-κB/α-tubulin ratio (**A**). Data were normalized to CON. Values are mean ± SEM (CON: *n* = 4; SNL: *n* = 4; 100CUR: *n* = 4; 50CMO: *n* = 4). * *p* < 0.050 vs. SNL ipsilateral leg. The representative blot images display NF-κB^Ser536^, NF-κB and α-tubulin for CON, SNL, 100CUR, and 50CMO groups (**B**). MW, molecular weight (kDa).

A significant main group effect was observed for NF-κB^Ser536^/NF-κB/α-tubulin (i.e., NF-κB activation) in the plantaris. In the plantaris, the SNL (2.40 ± 0.39) group had greater NF-κB activation compared to the CON (1.00 ± 0.29; *p* = 0.021), the 100CUR (0.61 ± 0.10; *p* = 0.003), and the 50CMO (0.77 ± 0.25; *p* = 0.009) group (Figure 3). In addition, the 100CUR (0.61 ± 0.10) group had lower NF-κB activation compared to the CON (1.00 ± 0.29; *p* = 0.046) group (Figure 3). No significant differences were observed for NF-κB protein content between the ipsilateral leg and the contralateral leg in all groups in the plantaris muscle.

#### 3.2.4. Interleukin-1β (IL-1β)

**Figure 4:**
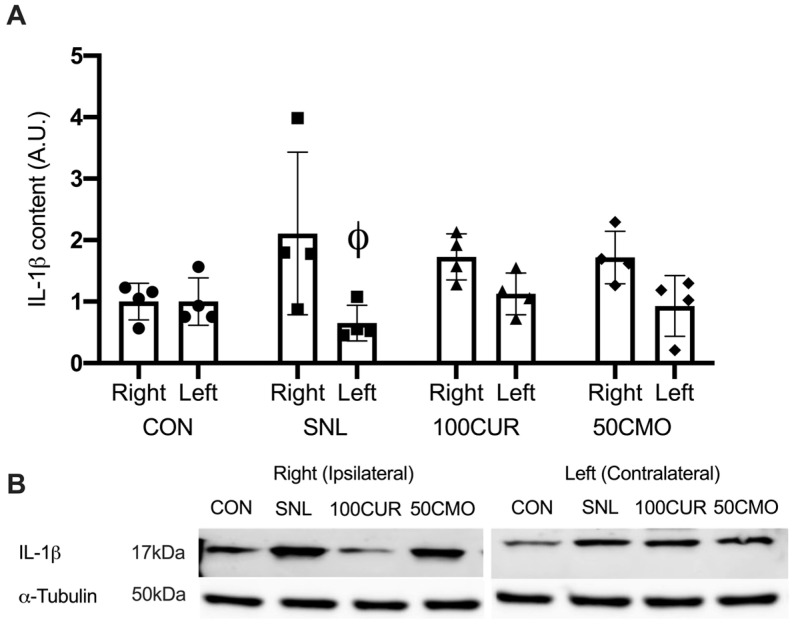
Plantaris protein content results for interleukin-1β (IL-1β) (**A**). Data were normalized to CON. Values are mean ± SEM (CON: *n* = 4; SNL: *n* = 4; 100CUR: *n* = 4; 50CMO: *n* = 4). Φ *p* < 0.050 vs. ipsilateral leg. The representative blot images display IL-1β and α-tubulin for CON, SNL, 100CUR, and 50CMO groups (**B**). MW, molecular weight (kDa).

A significant group x leg interaction effect was observed for IL-1β protein content such that the ipsilateral leg (2.11 ± 0.66) was greater than the contralateral leg (0.65 ± 0.14; *p* = 0.041) in the SNL group, whereas no significant differences were observed between ipsilateral and contralateral leg in CON, 100CUR, and 50CMO groups in the plantaris muscle (Figure 4).

## 4. Discussion

To our knowledge, this study is the first to investigate the effects of curcumin and bisdemethoxycurcumin on CaMKII and NF-κB activation, IL-1β, and muscle cross-sectional area (CSA) in spinal nerve ligation (SNL) rodent skeletal muscle. Consistent with previous studies [61,62,63,64], 4 weeks following SNL surgery resulted in muscle atrophy in the tibialis anterior (TA) muscle compared to the control group with no differences in the size of the soleus muscle. Notably, attenuated CSA reduction was attenuated in SNL rats supplemented with 100 mg curcumin/kg BW but not with 50 mg bisdemethoxycurcumin/kg BW (50CMO). Furthermore, in the plantaris muscle, CaMKII activation was greater following SNL surgery compared to CON, 100CUR, and 50CMO and compared to the contralateral leg. NF-κB activation was greater following SNL surgery compared to the control, 100CUR, and 50CMO but was not different between the ipsilateral and contralateral leg. Despite the greater NF-κB activation in both legs, IL-1β was greater in the ipsilateral leg compared to the contralateral leg in the SNL group. Together, the observed reduction in Ca^2+^ signaling and inflammation in type II plantaris muscle fibers might reflect the changes within the type II TA muscle fibers which may contribute to the mitigation of TA mass loss with curcumin supplementation.

### 4.1. Muscle Fiber Cross-Sectional Area

A reduction in muscle CSA was observed in the TA muscle but not in the soleus muscle in the SNL group compared to CON. This finding is in contrast to previous studies which observed soleus atrophy following denervation [65,66]. The discrepancy could be due to the different nerve damaging protocols. This study employed a loose ligation technique (axons intact), whereas previous studies used crush injuries or spinal nerve transection methods that cause more severe nerve damage in animals [65,66]. Consequently, the severity of denervation induced by this current protocol is expected to be lower than that of a crush injury or spinal nerve transection [67]. However, muscle atrophy of the TA is consistent with previous studies [61,62,63,64]. While denervation models consistently induce muscle atrophy across various muscles, affecting both type I and type II fibers [5,65,66,68,69], muscle mass loss due to denervation occurs more rapidly in type II fibers than in type I fibers [70,71]. While the mechanism of preferential muscle mass loss in type II fibers is not known, Chin et al. suggested that a compensatory adaptive response involving the upregulation of CaMKII splice variants to counter the ongoing muscle atrophy appears to exist only in the type I but not type II muscle [72]. Curcumin has been used to alleviate symptoms of denervation (e.g., peripheral neuropathic pain) [24,39,40] and attenuate TA muscle mass loss in nerve damage-induced denervation models [24,25]. Consistent with previous studies that administered curcumin intravenously (0.2 mg/day) over a 4-week period [24,25], daily oral supplementation of 100 mg/kg BW of curcumin but not 50 mg/kg BW bisdemethoxycurcumin attenuated TA muscle mass loss following SNL. Interestingly, sex may play a role in the severity of muscle atrophy experienced following SNL. Given the well-known protective effect of estrogen, female rats tend to experience preserved muscle mass during muscle atrophy as shown following inactivity compared to male rats [73,74]. Future investigations are needed to compare the effects of curcumin and bisdemethoxycurcumin across various denervation models that produce different severities of muscle atrophy in both male and female rats.

### 4.2. AChR Content

Denervation-induced muscle size loss has, at least partly, been attributed to CaMKII activation and the pro-inflammatory response such that inhibition of either CaMKII or NF-κB activity prevents atrophy [18,19]. Due to limited TA muscle samples, plantaris muscle samples were collected as a surrogate to measure denervation-induced molecular changes. Both the TA and plantaris are innervated by the L5 sciatic nerve and are composed primarily of type II muscle fibers [58]. Following denervation, increased AChR turnover [75,76,77] leads to the integration of highly Ca^2+^ permeable AChR [27,28,29,30] which may lead to an increase in sarcoplasmic Ca^2+^ [31]. The absence of change in AChR protein following SNL could partly be attributed to an overall increase in both AChR degradation and AChR synthesis [75,76,77]. Indeed, the expression of AChR subunits has been shown to be upregulated within 7 days following denervation [75,78,79]. While the total AChR protein was not different among groups, we speculate that the increase in sarcoplasmic Ca^2+^ influx through increased AChR turnover may partly explain the greater CaMKII activation found in the current study and previous studies [10,18].

### 4.3. CaMKII Activation, NF-κB Activation, and IL-1β

CaMKII activation of the ipsilateral leg only indicates that denervation results in attenuated Ca^2+^ signaling of the denervated muscle potentially without influencing Ca^2+^ signaling of the contralateral leg. This finding is supported by the hypothesis that denervation induces increased sarcoplasmic Ca^2+^ of the effect tissue but not systemically [17]. Activated CaMKII stimulates muscle atrophy directly by increasing proteasome protein expression (i.e., muscle RING-finger protein-1 and atrogin1) [18] and indirectly by activating NF-κB [33]. NF-κB activation leads to a pro-inflammatory state (i.e., IL-1β) [80,81,82] which could result in muscle atrophy [19]. In line with this, the SNL group demonstrated greater NF-κB activation alongside increased CAMKII activation compared to CON,100CUR, and 50CMO, with no difference in NF-κB activation of the ipsilateral leg compared to the contralateral leg. While the mechanism is not known, one speculation is that cytokines released from the ipsilateral leg (e.g., tumor necrosis factor-alpha) [83] could activate NF-κB in the contralateral leg [84]. However, these cytokines may not lead to an increase in IL-1β in the contralateral leg.

Concomitantly, curcumin and bisdemethoxycurcumin supplementation attenuated CaMKII activation and promoted anti-inflammatory activity (i.e., lower NF-κB activation and IL-1β) and were attenuated in the plantaris muscle. Although the specific mechanism by which curcumin affects muscle mass loss has not been investigated, studies have shown that curcumin reduces CaMKII activation in neuronal cells in a dose-dependent manner [26,44,45,46]. Additionally, curcumin has been found to decrease NF-κB activation, along with reducing IL-1β protein levels in pro-inflammatory lung tissue [49]. Given the role of CaMKII and NF-κB activations in SNL-induced muscle atrophy, we speculate that the observed reduction in Ca^2+^ signaling and inflammation in type II plantaris muscle fibers might reflect the changes within the type II TA muscle fibers which may contribute to the mitigation of TA mass loss with curcumin supplementation.

Similar to curcumin, bisdemethoxycurcumin has been shown to reduce NF-κB expression in renal and immune cells following bisdemethoxycurcumin supplementation [47,48]. Indeed, curcumin and bisdemethoxycurcumin exhibited an anti-inflammatory effect (i.e., attenuated increase in IBA-1, CD11b, and GFAP gene expression) in the spinal cord and amygdala observed in previously published data from the same study [57]. However, in contrast to curcumin, bisdemethoxycurcumin supplementation did not attenuate an SNL-induced reduction in muscle CSA despite its attenuated CaMKII activation and promoted anti-inflammatory effect. While both curcumin and bisdemethoxycurcumin demonstrate anti-inflammatory effects, in comparison to curcumin, bisdemethoxycurcumin suppressed NF-κB activity to a lesser extent [49]. Santosh et al. speculated that the phenyl methoxy groups in curcumin play a critical role in suppressing NF-κB activation which are absent in bisdemethoxycurcumin [49]. Given the role of NF-κB in muscle atrophy, mitigated suppression of NF-κB activity by bisdemethoxycurcumin could, at least partly, contribute to the sustained reduction in muscle CSA compared to curcumin.

### 4.4. Limitations

While our results indicated that curcumin (100 mg/kg BW) supplementation effectively attenuated TA muscle size loss following SNL, bisdemethoxycurcumin (50 mg/kg BW) supplementation did not yield the same effect. Both supplements, however, reduced CaMKII and NF-κB activations and IL-1β content, even though the groups differed in supplementation dosages. A lower dose of bisdemethoxycurcumin was selected based on the findings from previous studies which demonstrated greater chemical stability [51] and bioavailability [52] of bisdemethoxycurcumin compared to curcumin. Furthermore, based on previously published data from the same study, 50 mg/kg BW of bisdemethoxycurcumin had a similar decrease in mechanical pain hypersensitivity compared to 100 mg/kg BW of curcumin. However, 50 mg/kg BW of bisdemethoxycurcumin and 100 mg/kg BW of curcumin at anti-atrophic effect did not result in a similar anti-atrophic effect. A small sample size from the current pilot study could contribute to the lack of significant difference observed in muscle CSA despite a 60% larger CSA with bisdemethoxycurcumin supplementation compared to the SNL group. On the other hand, muscle atrophy is a complex phenomenon that involves multiple organelles, including the mitochondria [85]. Dysregulation of mitophagy is one factor which contributes to mitochondrial dysfunction during muscle atrophy [85]. Previous studies have demonstrated a protective effect of curcumin on the mitochondria [86,87]. Indeed, curcumin has been shown to regulate mitophagy and preserve mitochondrial function following injury in neuronal cells [88,89]. Mitigated mitochondrial dysfunction could contribute to the greater attenuation of muscle atrophy by curcumin compared to bisdemethoxycurcumin. Future studies are needed to compare the effect of curcumin and bisdemethoxycurcumin supplementation on alternative factors such as mitochondrial dysfunction contributing to muscle atrophy in denervation models.

### 4.5. Translational Implications

Denervation-induced muscle atrophy is a significant clinical challenge. This study highlights the translational potential of curcumin and bisdemethoxycurcumin as therapeutic agents for this condition by attenuating denervation-induced muscle mass loss. In addition, curcumin and bisdemethoxycurcumin are recognized for their safety [90], which makes them suitable candidates for clinical use. Based on the allometric scaling principle [91], 100 mg/kg BW curcumin supplementation corresponds to ~600 mg of curcumin in a 70 kg individual. A 600 mg dose of curcumin is readily available and thus feasible for individuals to incorporate into their nutrition regiment.

## 5. Conclusions

In summary, SNL resulted in a greater Ca^2+^ signaling (i.e., CaMKII activation) and inflammation (i.e., NF-κB activation and IL-1β) compared to CON, but these changes were attenuated with curcumin and bisdemethoxycurcumin supplementation. Curcumin, but not bisdemethoxycurcumin, was able to attenuate SNL-induced muscle size loss. The observed reduction in Ca^2+^ signaling and inflammation in type II plantaris muscle fibers might reflect the changes within the type II TA muscle fibers which may contribute to the mitigation of TA mass loss with curcumin supplementation.

## Data Availability

The raw data supporting the conclusions of this article will be made available by the authors on request.

## Supplementary Materials

The following supporting information can be downloaded at: https://www.mdpi.com/article/10.3390/cimb46110742/s1, Figure S1: Representative full Western blot images.
