# Supplementary material for: Curcumin Mitigates Muscle Atrophy Potentially by Attenuating Calcium Signaling and Inflammation in a Spinal Nerve Ligation Model
Source: Curr Issues Mol Biol. 2024 Nov 5;46(11):12497–511. doi: 10.3390/cimb46110742 (PMC11592774; doi:10.3390/cimb46110742)
Supplement: Supplementary file 1 [file cimb-46-00742-s001.zip › cimb-3267650-supplementary.pdf]

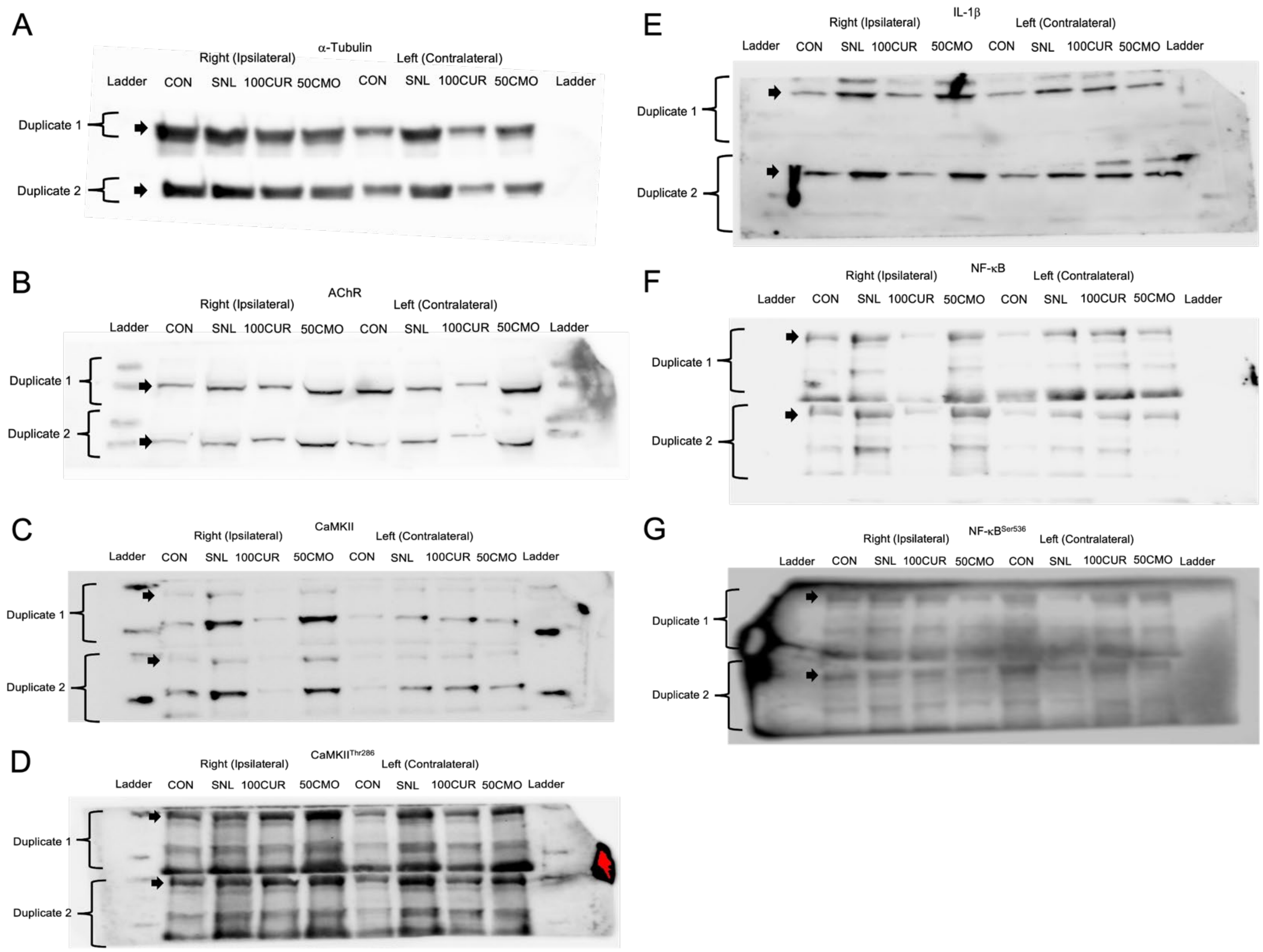

Supplemental Figure S1. Representative Full Western Blot Images. α-Tubulin (A), AChR (B), CaMKII (C), CaMKII^{Thr286} (D), IL-1β (E), NF-κB (F), NF-κB^{Ser536} (G).
